# Chloramphenicol Derivatization in Its Primary Hydroxyl Group with Basic Amino Acids Leads to New Pharmacophores with High Antimicrobial Activity

**DOI:** 10.3390/antibiotics12050832

**Published:** 2023-04-29

**Authors:** Artemis Tsirogianni, Georgia G. Kournoutou, Maria Mpogiatzoglou, George Dinos, Constantinos M. Athanassopoulos

**Affiliations:** 1 Synthetic Organic Chemistry Laboratory, Department of Chemistry, University of Patras, GR-26504 Patras, Greece; a.tsirogian@upnet.gr; 2 Department of Biochemistry, School of Medicine, University of Patras, GR-26504 Patras, Greece; gkurnutu@upatras.gr (G.G.K.);

**Keywords:** antibiotic resistance, antibiotics, chloramphenicol, peptidyl transferase, ribosome

## Abstract

In a previous study published by our group, successful modification of the antibiotic chloramphenicol (CHL) was reported, which was achieved by replacing the dichloroacetyl tail with alpha and beta amino acids, resulting in promising new antibacterial pharmacophores. In this study, CHL was further modified by linking the basic amino acids lysine, ornithine, and histidine to the primary hydroxyl group of CHL via triazole, carbamate, or amide bonding. Our results showed that while linking the basic amino acids retained antibacterial activity, it was somewhat reduced compared to CHL. However, in vitro testing demonstrated that all derivatives were comparable in activity to CHL and competed for the same ribosomal binding site with radioactive chloramphenicol. The amino acid–CHL tethering modes were evaluated either with carbamate (**7, 8**) derivatives, which exhibited higher activity, or with amide- (**4**–**6**) or triazole-bridged compounds (**1**–**3**), which were equally potent. Our findings suggest that these new pharmacophores have potential as antimicrobial agents, though further optimization is needed.

## 1. Introduction

**Figure 1:**
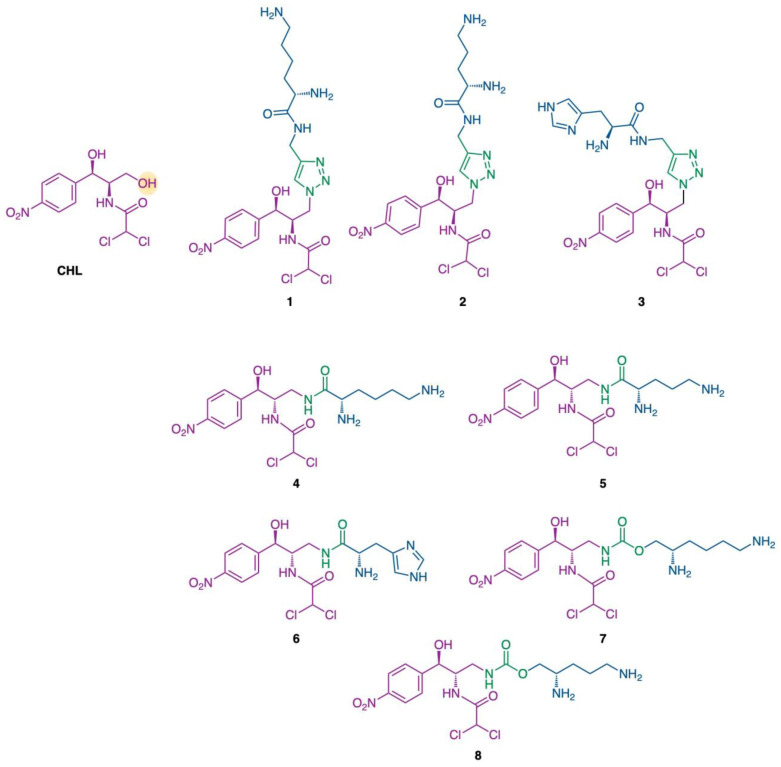
Structures of the CHL derivatives encountered in this work. Pink represents the mother molecule CHL, orange the modified prime hydroxyl group, green the linkers, and blue the basic amino acids lysine (**1**, **4**, **7**), ornithine (**2**, **5**, **8**), and histidine (**3**, **6**).

Antibiotic resistance is a major problem for therapeutic medicine, although this phenomenon is as old as the appearance of antibiotics in the living world [1]. Isolated caves and permafrost sediments have shown that resistance is not the result of the relatively recent use of antibiotics, but rather the non-stop competition for resources among microorganisms [1,2]. Today, two basic methods exist to address this critically rising antibiotic resistance: a vaccine or medicine. Keeping in mind that vaccination in this area is only recently being successfully explored, a vaccine has been developed only for one of the leading pathogens up to now, *S. pneumoniae* [3]. In reality, it will take a long time to reach vaccine sufficiency for the rest of the most dangerous pathogens (*Enterococcus faecium*, *Staphylococcus aureus*, *Klebsiella pneumoniae*, *Acinetobacter baumannii*, *Pseudomonas aeruginosa*, and *Enterobacter species*) [4]. Therefore, the derivatization of known antibiotics in the production of safe and effective antibiotics is a one-way road [5,6,7]. Following the successfully established procedure here, we present the chemical synthesis and evaluation of new (CHL) chloramphenicol-pharmacophores that tether the basic amino acids lysine, ornithine, and histidine to the CHL C3 position using three different bridges: amide, triazolium, or carbamate (Figure 1, green color). Earlier research with basic amino acids tethered to CHL demonstrated that these compounds increased their ribosome affinity and severely inhibited ribosome function [8,9]. Chloramphenicol (CHL), first released in 1947 [10], is a broad-spectrum antibiotic that acts on ribosomes and is effective against a variety of Gram-positive and Gram-negative bacteria. The medicine quickly gained popularity when it was approved for clinical use due to its inexpensive cost, efficacy, and the limited number of moderate adverse effects. However, soon after, its initial minor adverse effects developed into significant conditions, including aplastic anemia and bone marrow depression [11,12]. As a result, the indications for prescribing chloramphenicol were reduced due to the drug’s toxicity and the discovery of safer substitute antibiotics, and its usage was eventually discontinued. Although CHL was medically devalued, it never lost its appeal as a tool for research into protein synthesis, particularly ribosome structure and function. It has been determined through several structural and functional studies that CHL binds in the large ribosomal subunit, partially obscuring the A-site substrate’s binding surface and impeding protein synthesis [13,14,15]. More precisely, the aromatic ring of the ribosome-bound CHL (Figure 1) overlaps with the placement of side chains of the incoming aa-tRNAs, thus efficiently preventing the aminoacyl moiety of aa-tRNA from properly accommodating into the peptidyl transferase center (PTC) active site [16]. This model has recently been revised based on novel data that support the theory that CHL acts as a context-specific inhibitor of translation whose action depends on the nature of specific amino acids in the nascent chain and the identity of the residue entering the A-site. More precisely, chloramphenicol-mediated inhibition is stimulated when a nascent peptide in the ribosome carries an alanine amino acid in the penultimate position and preferentially aspartic acid and lysine in the P-site and A-site, respectively [17,18].

Studies on derivatizing chloramphenicol to improve its antibacterial effectiveness and reduce its harmful side effects were initiated early on because of the advent of undesired side effects and rising antimicrobial resistance. Numerous derivatives have been designed and produced up to this point, but none have been found to be better than the original chloramphenicol molecule [8,15,19,20,21,22]. Despite the derivatization approach’s lack of success to date, efforts have not stopped because it is one of the most fruitful ways of creating new-generation antibiotics to combat pathogenic resistance. Our findings indicate that linking of the three basic amino acids maintained antibacterial activity, albeit to a lesser extent, while in vitro assays revealed that all derivatives were comparably active to CHL and additionally competed with radioactive chloramphenicol for the same ribosomal binding site. Antimicrobial activity was optimized when tethering them through a carbamate bridge, while in vitro activity was a bit higher compared to CHL. That means there is still room to further improve these derivatives and use them as potential antimicrobial pharmacophores.

## 2. Results

### 2.1. Chemical Synthesis

**Scheme 1:**
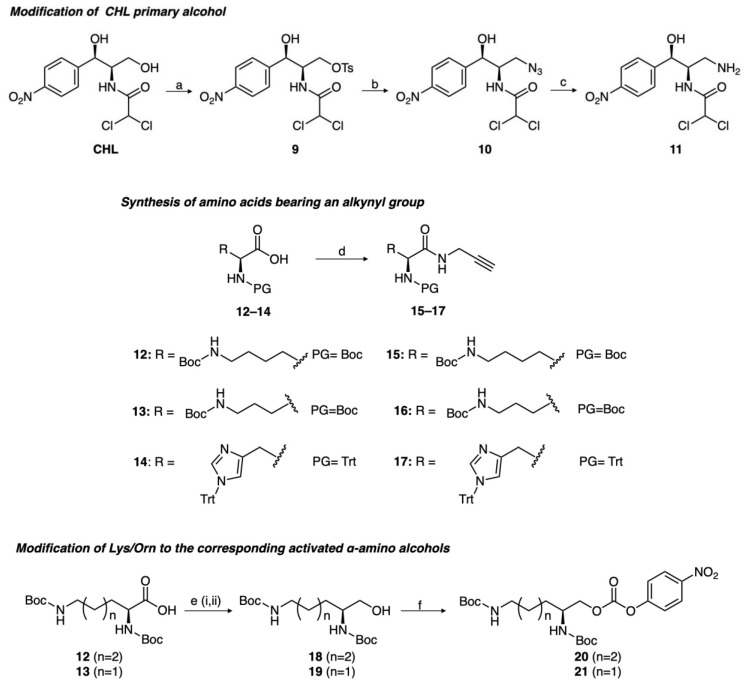
Synthetic pathways for intermediates **9**–**21**. Reagents and conditions: (a) TsCl, Et_3_N, THF, DMAP, 0 °C to RT, 24 h, 80%; (b) NaN_3_, DMF, 100 °C, 24 h, 77%; (c) Ph_3_P, THF/H_2_O, RT, 24 h, 70%; (d) propargylamine, HBTU/DIPEA, DCE, 0 °C to RT, 24 h, 65–70%; (e) (i) Et_3_N, ethyl chloroformate, THF; (e) (ii) NaBH_4_, MeOH, 0 °C to RT, 4 h, 77–80%; (f) 4-nitrophenyl chloroformate, DCE, Et_3_N, RT, 24 h, 82–84%.

The assembly of the various building blocks is described in Scheme 1. Regarding the CHL modification, commercially available CHL was treated with 4-toluenesulfonyl chloride (TsCl) in the presence of triethylamine (Et_3_N) and 4-dimethylaminopyridine (DMAP), followed by the addition of NaN_3_ to produce the azide **10**. The latter was then reduced to the corresponding primary amine **11**. On the other hand, the synthesis of the amino acid building blocks containing an alkynyl group (**15**–**17**) was accomplished by coupling the readily available *Boc-* or *Trt-* protected amino acids and propargylamine using the system HBTU/DIPEA. Moreover, activation and subsequent reduction of the carboxylic group of *N*-Boc-protected lysine and ornithine gave the corresponding primary alcohols **18** and **19**, which were then activated using 4-nitrophenyl chloroformate in the presence of triethylamine to obtain the corresponding carbonates **20** and **21**.

**Scheme 2:**
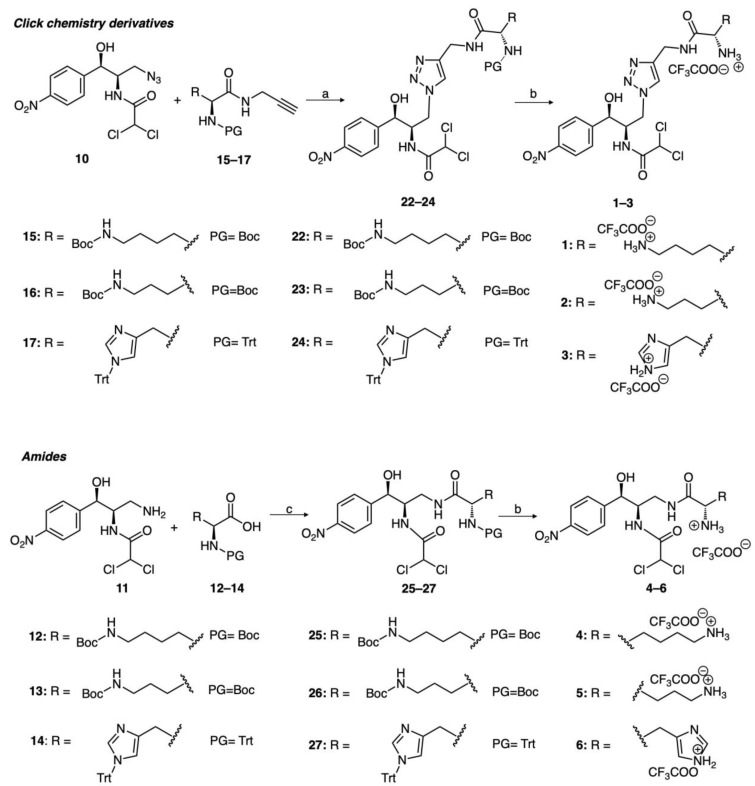

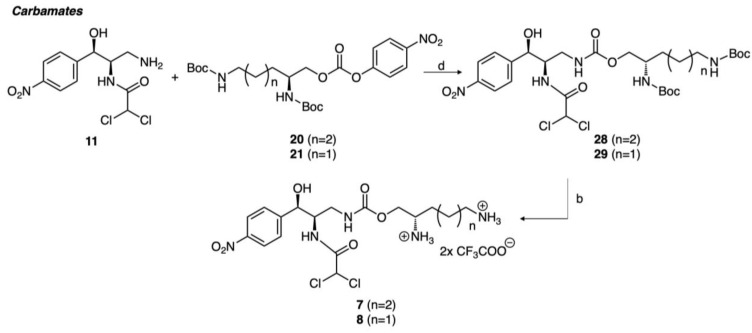
Synthetic pathways for compounds **1**–**8**. Reagents and conditions: (a) CuSO45H2O, sodium ascorbate, DCM/H2O 1:1, RT, 24 h,65–70%; (b) 50% TFA, TFE, DCM, 0 °C to RT, 4 h, 77–80%; (c) HBTU/DIPEA, DCE, RT, 24 h, 75–82%; (d) DIPEA, DCE, 0 °C to RT, 24 h, 78–86%.

The synthesis of various derivatives containing a triazole linker, CHL–amino acid amides, and CHL–amino acid carbamates is described in Scheme 2. The desired derivatives containing a triazole linker (**1**–**3**) were obtained through click chemistry involving the CHL–azide **10** and the *N*-protected amino acids bearing an alkynyl group (**15**–**17**), followed by TFA-mediated *N*-deprotection. The synthesis of CHL–amino acid amides (**4**–**6**) was achieved through coupling of the CHL–amine **11** with the *N*-protected amino acids **12**–**14** using the HBTU/DIPEA system, followed by TFA-mediated *N*-deprotection. The CHL–amino acid carbamates (**7, 8**) were subsequently synthesized through coupling of compound **11** with the activated carbonates **20** and **21,** followed by TFA-mediated *N*-deprotection.

### 2.2. Antibacterial Activity

**Figure 2:**
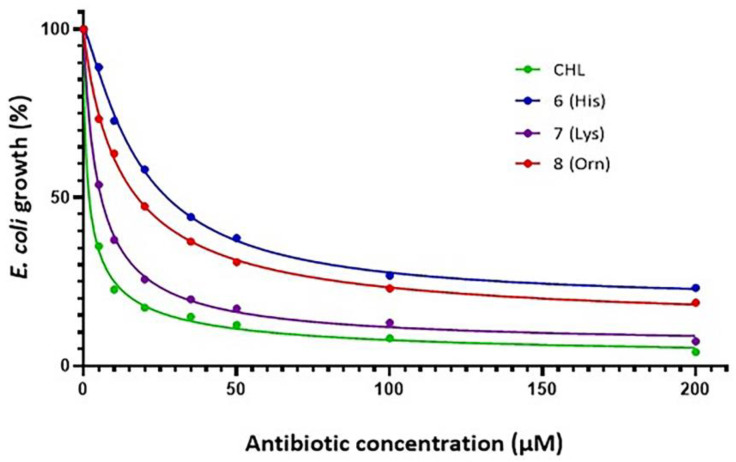
Bacteria *E. coli* ΔTolC growth in the presence of CHL and selected derivatives. Antibiotic concentrations were increased from 0.05 μM up to 200 μM. The calculated EC_50_ values are presented in Table 1.

All synthesized compounds initially underwent in vivo screening with the *E. coli* ΔTolC strain. Following culture growth with increasing antibiotic concentrations, the EC_50_ concentrations were determined, which represent the required concentration for the half-maximal effect of the culture and which were calculated as described in the Materials and Methods section. Growth inhibition as a function of concentration is represented in Figure 2, and the calculated EC_50_ values are represented in Table 1. As it is shown in Table 1, in contrast to the previous publication where the same amino acids had replaced the dichloroacetyl tail, antimicrobial activity now exists for almost all derivatives, although to a moderate level compared to the mother molecule CHL. The amino acid lysine, which is linked with a carbamate bridge, was the most active of all, with ornithine being more active than histidine. The bridges triazolium and amide showed an increased EC_50_ value, almost an order of magnitude greater than carbamate, which exhibited the highest activity. To further explore antimicrobial activity, *S. epidermidis* was used as a model Gram-positive pathogen and EC_50_ was calculated with almost similar results (data not shown). Next, all the compounds were tested in vitro. 

### 2.3. Protein Synthesis Inhibition

#### 2.3.1. Cell-Free Transcription–Translation Inhibition

First, the effect of the new compounds was tested on overall protein synthesis using a lysate-based cell-free transcription–translation experimental system from *E. coli* strain B [23], which was achieved by using the *Renilla reinformis* luciferase gene as a template [24]. This assay is one of the most physiological in vitro systems for protein synthesis currently available and is appropriate for the evaluation of translating inhibitors [25]. In each assay, luminescence was measured and was expressed as percent of control (without inhibitor).

**Figure 3:**
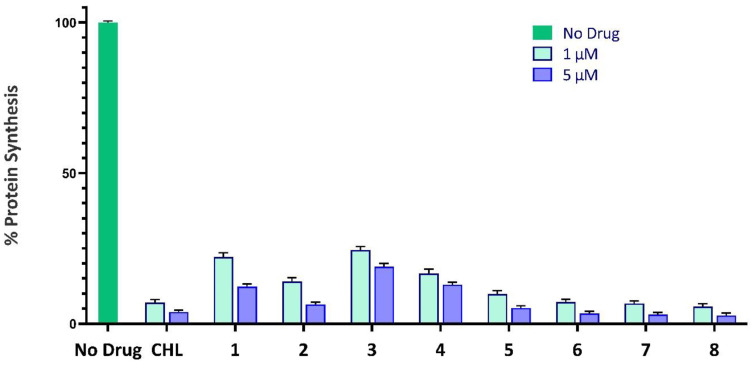
In vitro transcription–translation system in the presence of CHL derivatives (**1**–**8**). All compounds were tested in 1 μΜ and 5 μM concentrations and the results are presented as the percentage of control (without inhibitor).

**Figure 4:**
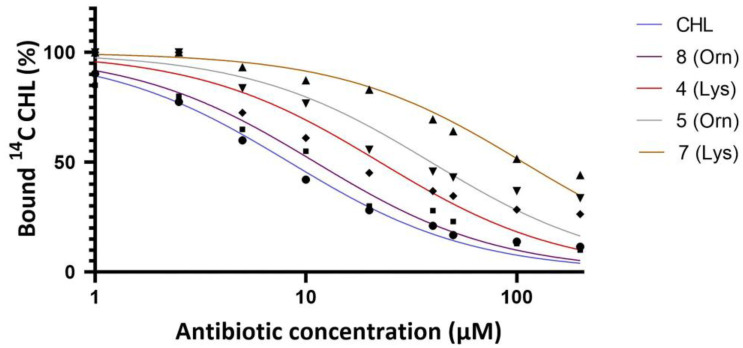
Competition in [^14^C]-CHL binding to vacant reassociated 70S ribosomes by non-radioactive CHL or CHL derivatives. Data are presented as the percentage of bound radioactive CHL in the absence (100%) and in the presence of antibiotics versus the antibiotic concentration. (•) Chloramphenicol, (■) compound **8**, (♦) compound **4**, (▼) compound **5**, (▲) compound **7**.

According to Figure 3, all derivatives were strong inhibitors of the synthesizing machinery, with similar potency to the mother molecule. That means the new compounds all keep the basic structure of the chloramphenicol pharmacophore with mild differentiation in terms of binding and inhibiting ribosomal function. To further explore their binding site and affinity for the ribosome, radioactively labeled chloramphenicol was used in increasing amounts to study their competition, though this was not labeled antibiotic concentration. As it is shown in Figure 4, there is competition for [^14^C] chloramphenicol binding, supporting the conclusion that all compounds share either the same overlapping sites or at least partially occupy them. Figure 4 does not show all experimental data to avoid plots overlapping. From the same figure, IC_50_ inhibition concentration was measured and the Ki values were calculated [26] for all the tested compounds, which are presented in Table 2. 

#### 2.3.2. Puromycin Reaction

**Figure 5:**
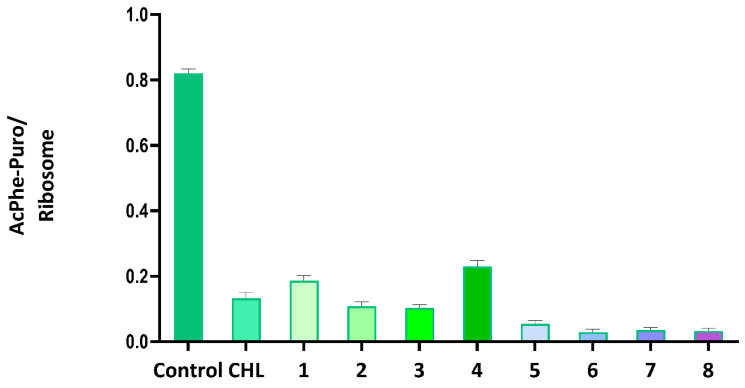
AcPhe-puromycin formation in the absence (control) and in the presence of 10 μM of each antibiotic. Post-translation ribosomal complex carrying deacylated tRNA_f_^Met^ in the E-site and Ac[^3^H] Phe-tRNA at the P-site of MF-mRNA-programmed 70S ribosomes was reacted with 1 mM puromycin at 37 °C for 2 min in the absence (control) and in the presence of antibiotics.

The puromycin reaction was also carried out to further explore the functionality of the binding site occupied by antibiotics and their mode of action. It is known that puromycin is a pseudosubstrate that binds in the A-site mimicking aminoacyl-tRNA and forms a peptide bond (peptidyl-puromycin) with peptidyl-tRNA bound in the P-site [27]. Moreover, puromycin reacts only with the P-site-bound peptidyl-tRNA and not with A-site-bound peptidyl-tRNA. Therefore, it is a tool for discrimination of the site of peptidyl-tRNA binding, except for the kinetic measurement of peptidyl transferase activity, the catalytic ribosomal center responsible for peptide bond formation. Additionally, CHL occupies the ribosomal A-site and inhibits the puromycin reaction [26]. According to Figure 5, all derivatives strongly inhibit the puromycin reaction, suggesting that all compounds occupy the A-site, inhibiting either puromycin binding or its accommodation.

## 3. Discussion

**Figure 6:**
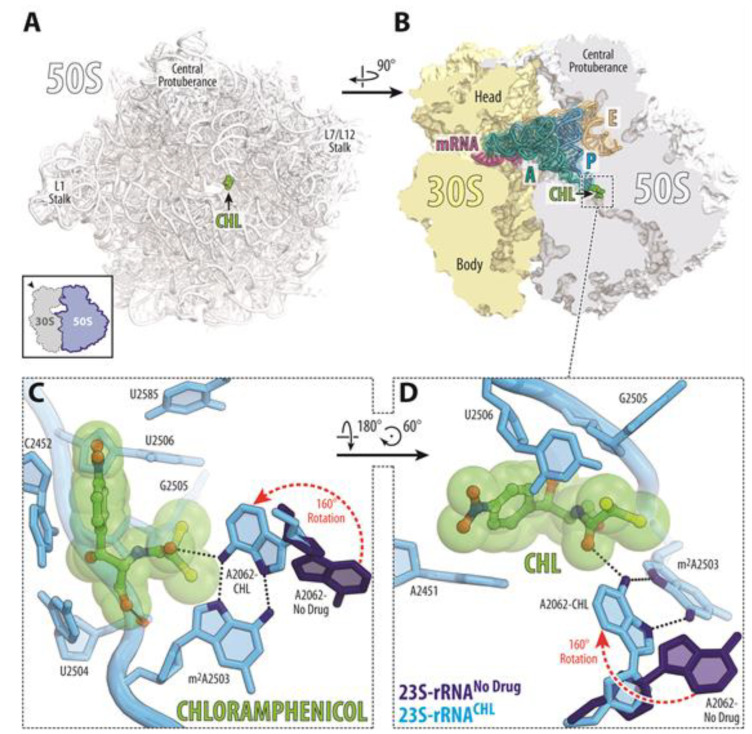
Structure of CHL in complex with programmed 70S ribosomes and A-, P-, and E-site tRNAs. (**A**,**B**) Overview of the CHL binding site (green) in the *Thermus thermophilus* (Tth) 50S ribosome viewed from the PTC down the tunnel, as indicated by the inset (**A**), or as a cross-cut section through the 70S ribosome (**B**). The 30S subunit is shown in light yellow, the 50S subunit is in light blue, the mRNA is in magenta, and the A-, P-, and E-site tRNAs are colored green, dark blue, and orange, respectively. (**C**,**D**) Close-up views of the CHL bound in the PTC, PDB entry 6ND5 [16]. *E. coli* nucleotide numbering is used. Potential H-bond interactions are indicated with dashed lines for the keto oxygen and solid lines for the prime -OH. Note that rotation of A2062 is forming a H-bond after Hoogsteen base pairing with the m^2^A2503 of the 23S rRNA (red dashed arrow). The N6 and N7 atoms of nucleotides A2062 and m^2^A2503 are highlighted in dark blue. The unrotated conformation of A2062 observed in the absence of the drug is shown in dark blue (PDB entry 4Y4P [29]).

Our findings suggest that modification of CHL’s primary hydroxyl group and subsequent tethering with lysine, ornithine, or histidine provided derivatives that still retain considerable antibacterial activity, though at a lower level than the original molecule. Among the three basic amino acids, lysine demonstrated higher in vivo activity compared to ornithine and histidine. However, all three amino acids showed similarly high or higher activity compared to CHL in vitro. Additionally, competition for binding with radioactive chloramphenicol (as shown in Figure 4) demonstrates that the new compounds bind at the same ribosomal site or partially occupy the previously determined chloramphenicol binding site [13,14]. This site was further confirmed through high-resolution crystal structure data (as seen in Figure 6) from recent studies [16]. According to these studies (Figure 6), the tail of the aromatic ring adopts a unique conformation due to stabilization provided by the H-bond formed with the nucleotide A2062 of the 23S rRNA. This base rotates by ~160° around its N-glycosidic bond into a position where it forms a H-bond between the keto oxygen of CHL and the N6 atom of A2062. Another H-bond also exists between the primary -OH group of CHL and the phosphate group of G2505 (Figure 6C) [16]. This bond is missing in the new compounds because the primary -OH has been replaced. Moreover, the fact that the new compounds are still strongly bound to the ribosome (see low Ki constants, Table 2) supports the possibility for a new H-bond, considering that all inserted groups contain a nitrogen atom which can also offer an uncoupled electron pair; however, this remains to be elucidated. Furthermore, the rotation of base A2062, which occurs in the presence of CHL, is uncertain in the presence of our derivatives. However, it is suggested that this may still occur because binding of not only CHL, but also erythromycin, causes the same characteristic rotation of A2062 to thus form a Hoogsteen base pair with m^2^A2503, as observed in the case of CHL [16]. According to recent crystallographic data with different peptidyl-tRNAs bound on the ribosome, the CHL binding site is formed not only by the ribosome alone but also by the growing polypeptide, and the shape of the drug-binding pocket continuously changes as the nascent chain is created by the ribosome [28]. This can easily explain why chloramphenicol and its derivatives are more effective in an in vitro transcription–translation system compared to their Ki values as they were calculated on vacant ribosomes (Table 2). From this viewpoint, the nascent peptide plays the role of a drug-affinity modulator [18] and should be considered as a part of the CHL binding site. That is why Mankin and colleagues suggest that chloramphenicol should be considered as an uncompetitive, rather than competitive, ribosome inhibitor. Uncompetitive inhibitors bind adjacent to the active site of an enzyme and require a substrate to be present before binding [28]. Based on this recent theory of CHL’s mode of action, our derivatives could also be considered as uncompetitive ribosome inhibitors, but this new suggestion must be proved.

Another important question concerns the success of the linker connecting CHL’s C3 position with basic amino acids and its contribution to the derivative’s biological effects. According to the results, the carbamate is more effective concerning triazolium and/or amide, depending perhaps on the number of available hydrogen bonds which could take place in the new accommodation. The length of the replacing group is also another issue of interest but considering that the three inserted basic amino acids have around the same length, all can be accommodated in a successful way around the peptidyl transferase center. In conclusion, our new compounds offer new dynamic pharmacophore structures with room for further modifications and development. Our next goal will be the combination of these prime HO group modifications with previously inserted derivatizations in the dichloroacetyl tail [24] to improve our molecule’s characteristics as a promising antibacterial agent.

## 4. Materials and Methods

### 4.1. Chemical Synthesis

More detailed information on the synthesis and structural characterization of the synthesized derivatives can be found in the Supplementary Materials, which includes synthetic schemes and procedures, as well as ^1^H, ^13^C NMR, and ESI–MS spectra.

### 4.2. Bacterial Strains

The wild-type *E. coli* K12/MG1655 strain was used to isolate the components of the functional assays, such as ribosomes and tRNA, and the SQ110 ΔTolC variant [23] was used to test antibacterial activity. All strains were grown at 37 °C in LB medium with continuous shaking, and for the ΔTolC strain we also included 50 μg/mL kanamycin.

### 4.3. Biochemical Preparations

70S reassociated ribosomes free of endogenous tRNAs were prepared from *E. coli* K-12/MG1655 cells as described by Blaha et al. [30] and were kept in buffer containing 20 m MHEPES/KOH, 50 mM CH_3_COONH_4_, 6 mM (CH_3_COO)_2_Mg, and 4 mM mercaptoethanol (buffer A) at a pH of 7.6. MF-mRNA, encoding Met-Phe [sequence: GGG(A_4_G)_3_AAAAUGUUC(A_4_G)_3_AAAU] [31] was prepared according to Schafer et al. [32]. EF-G with C-terminal His-tags was isolated from *E. coli*, as described previously [25]. Ac[^3^H]Phe-tRNA was prepared using specific tRNA under standard conditions (Rheinberger et al., 1988 [33]) and was separated from uncharged tRNA by reverse-phase HPLC on a Nucleosil column using a programmed binary gradient of buffer 1 (20 mM ammonium acetate, pH 5.0, 10 mM magnesium acetate, 400 mM NaCl) and 2 (60% *v*/*v* methanol in buffer 1). 70S ribosomes (0.3 μM) were first incubated at 37 °C for 15 min with MF-mRNA (2.0 μM) and 0.6 μM uncharged tRNA phenylalanine initiator-phenylalanine specific (deacylated tRNA_f_^Met^ in order to prefill the P-site). Nonenzymatic binding was then performed, followed by the addition of appropriately charged Ac[^3^H]Phe-tRNA at a final concentration of 0.5 μM, which occupied the A-site. The complex named pre-translocation was then incubated with EF-G (final concentration 0.3 pmoles/pmol 70S) in the presence of GTP (0.12 mM) and was incubated at 37 °C for an additional 10 min. The post-translocation complex that was formed as described above carried a deacylated tRNA in the E-site and a peptidyl-tRNA in the P-site, resembled the elongating ribosome, and was isolated free of the unbound ligands (tRNAs, EF-G, GTP) via centrifugation through a 10% sucrose cushion at 65,000× *g* for 18 h at 4 °C. The pellet was diluted in buffer A and was used for the puromycin reaction.

L-[2,3,4,5,6-[^3^H]-phenylalanine was purchased from Amersham Pharmacia Biotech (Piscataway, NJ, USA), whereas [^14^C]-chloramphenicol was obtained from Perkin Elmer (Richmond, CA, USA). Specific tRNAs were purchased from Sigma.

### 4.4. Inhibition of Translation Using an E. coli-Based In Vitro Cell-Free Expression System

In this study, the S30 T7 high-yield protein expression system (Promega Corporation) was used. Screening assays of our compounds were performed in small-scale in vitro transcription/translation reactions, using the included control DNA template containing the *Renilla reniformis* luciferase gene as previously described [24]. From each reaction, 2.5 μL samples were taken and diluted by adding 97.5 μL of the lysis buffer from the Renilla Luciferase Assay kit (Biotium) used. The mix was thoroughly mixed and 50 μL was then placed into a 96-well white opaque plate (Greiner). Right before the measurement, 50 μL of the assay reagent was added to all the samples, which were then mixed and immediately placed in a luminometer (Perkin Elmer Victor2) for measurement. Data were presented as percent of control (without antibiotic).

### 4.5. EC_50_ Determination

Cells were grown overnight in Luria–Bertani medium and were diluted into fresh Luria–Bertani medium again and were grown. Early exponential-phase cultures were then diluted to a final absorbance at 600 nm equal to 0.050 and were incubated with each one of the antibiotics at indicated concentrations at 37 °C for the appropriate time to reach absorbance equal to 0.700 of the control sample, which was grown without antibiotics.

From dose–response curves, the half-maximal effective concentration (EC_50_) for each compound and strain was estimated. EC_50_ represents the molar concentration of a compound that produces 50% of the maximum possible effect. The EC_50_ values were mathematically determined by non-linear regression fitting of the observed culture optical density values (expressed as a percentage of 0.700 (*y*)) into the Hill equation,

$$y=min+\frac{max-min}{1+{\left(\frac{x}{EC_{50}}\right)}^{-n}}$$

where *min* and *max* are the lowest and highest observed values of the culture’s optical density, respectively, *x* is the concentration of the tested compound, and *n* is the Hill coefficient that represents the largest absolute value of the curve slope. *EC*_50_ is equal to the *x* value of the sigmoid’s midpoint. Fitting was performed using the Four Parameter Logistic Curve of Sigma Plot Version 11.0 (Systat Software, Inc., San Jose, CA, USA) for exact graphs and data analysis.

### 4.6. Competition in [^14^C]-Chloramphenicol Binding

Reassociated 70S ribosomes (0.20 μΜ final concentration) were incubated in buffer A [26] with [^14^C]-chloramphenicol (150 dpm/pmol) and increasing concentrations of radioactive CHL, to choose the best radioactive CHL concentration for competition experiments [26]. After incubation for 10 min at 37 °C, the mixture was diluted with 3 mL of cold buffer A and was filtered through a 25-mm in diameter cellulose nitrate membrane filter (Millipore, 0.45 μm pore size) [26]. The filter was immediately washed twice with 3 mL of cold buffer A and the bound radioactivity was determined by measuring the radioactivity bound on the filter in a liquid scintillation counter. Next, the binding of [^14^C]-chloramphenicol was studied in competition experiments with cold chloramphenicol or CHL derivatives by maintaining a constant concentration of [^14^C]-chloramphenicol (6 μM) and increasing concentrations of non-radioactive competitors.

### 4.7. Puromycin Reaction

The reaction between the previously prepared post-translocation ribosomal complexes and an excess of puromycin in buffer A was carried out at 37 °C for 2 min [34]. The ionic conditions were 20 mM HEPES-KOH (pH 7.6), 4.5 mM magnesium acetate, 150 mM ammonium acetate, 2 mM spermidine, 0.05 mM spermine, and 4 mM *β*-mercaptoethanol, which were kept constant throughout all the steps of complex formation and puromycin reaction. This buffer approximates physiological conditions with respect to the concentrations of the essential ions using NH_4_^+^ ions instead of K^+^ [30].

The reaction volume was 20 μL with a final puromycin concentration of 1 mM, in the absence or presence of 10 μM of each antibiotic. The reaction was stopped by the addition of an equal volume of 0.3 M sodium acetate saturated with MgSO_4_ at a pH of 5.5, with the resulting volume extracted using 1 mL of ethyl acetate and the radioactivity contained in 700 μL of the organic phase quantified by liquid scintillation. The results were expressed as the pmol ratio of acetyl-[^3^H]phenylalanine-puromycin per ribosome.

## Figures and Tables

**Table 1:** EC_50_ determination in μΜ and μgr/mL for all CHL derivatives.

Antibiotic	EC_50_ (μΜ)	EC_50_ (μgr/mL)
Chloramphenicol (CHL)	1.76	0.57
**1** (Lys)	25.06	8.10
**2** (Orn)	20.36	6.58
**3** (His)	49.21	15.90
**4** (Lys)	29.62	9.57
**5** (Orn)	18.58	6.03
**6** (His)	39.90	12.89
**7** (Lys)	5.01	1.62
**8** (Orn)	13.21	4.27

**Table 2:** Ki values determined according to the IC_50_ of each chloramphenicol derivative.

Antibiotic	K_i_ (μΜ)
Chloramphenicol	1.7
**1** (Lys)	3.8
**2** (Orn)	16.8
**3** (His)	12.3
**4** (Lys)	22.5
**5** (Orn)	8.3
**6** (His)	8.5
**7** (Lys)	4.7
**8** (Orn)	2.3

## Data Availability

Not applicable.

## Supplementary Materials

The following supporting information can be downloaded at: https://www.mdpi.com/article/10.3390/antibiotics12050832/s1, Scheme S1: Modifications of CHL’s primary alcohol group; Scheme S2: Synthesis of modified (*L*)-Lysine building blocks; Scheme S3: Synthesis of modified (*L*)-Ornithine building blocks; Scheme S4: Synthesis of modified (L)-Histidine building blocks; Scheme S5: Synthesis of derivatives **1**–**3**; Scheme S6: Synthesis of amides **4**–**6**, **25**–**27**; Scheme S7: Synthesis of carbamates **7**–**8**, **28**–**29**. Moreover, the section contains detailed synthetic procedures describing the preparation and the structural characterization by ^1^H, ^13^C NMR, and ESI–MS spectra of each compound synthesized for the purposes of this work.
