# Supplementary material for: Chloramphenicol Derivatization in Its Primary Hydroxyl Group with Basic Amino Acids Leads to New Pharmacophores with High Antimicrobial Activity
Source: Antibiotics (Basel). 2023 Apr 29;12(5):832. doi: 10.3390/antibiotics12050832 (PMC10215083; doi:10.3390/antibiotics12050832)
Supplement: Supplementary file 1 [file antibiotics-12-00832-s001.zip › antibiotics-2317427-supplementary.pdf]

SUPPORTING INFORMATION

General

All solvents were dried and/or purified according to standard procedures prior to use. Anhydrous Na_2SO_4 was used for drying solutions and the solvents were then routinely removed at ca. 40 ^{o}C under reduced pressure using a rotary vacuum evaporator. All reagents employed in the present work were commercially available and used without further purification. ^{1}H NMR spectra were obtained at 600.13 MHz and ^{13}C NMR spectra at 150.90 MHz on a Bruker AVANCEIII HD spectrometer. Chemical shifts (δ) are indicated in parts per million (ppm) upfield from TMS and coupling constants (J) are reported in hertz. ESI+ mass spectra were recorded at 30V, on a Micromass-Platform LC spectrometer using MeOH as solvent.

Melting points were determined with a Buchi SMP-20 apparatus and are uncorrected. When required, reactions were carried out under an inert argon atmosphere in pre-flamed glassware. Flash column chromatography (FCC) was performed on silica gel 60 (230-400 mesh), purchased by Merck and analytical thin layer chromatography (TLC) on Sigma-Aldrich silica gel 60F_{254} pre-coated aluminum foils (0.2 mm film). Spots on TLC plates were visualized using UV light at 254 nm and ninhydrine solution.

Experimental

A. Modifications of CHL's primary alcohol group

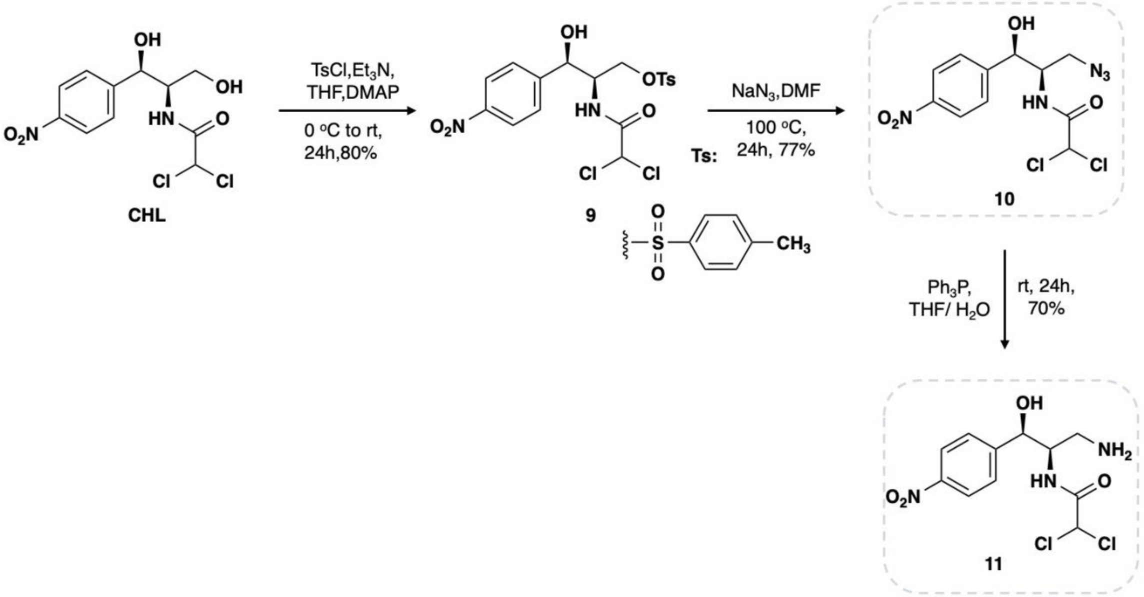

Scheme S1:Modifications of CHL's primary alcohol group

Synthesis of CHL-OTs (9)

A commercially available CHL (1.0 eq) was added to an ice-cold solution of THF (0.5 M) and Et_3N (1.2 eq) followed by a catalytic amount of DMAP. The reaction mixture was stirred at 0 ^{o}C for 10 min. After 10 min TsCl (1.0 eq) was added portion slowly, and the reaction mixture was stirred at room temperature overnight. The following day, the mixture was evaporated to dryness under vacuum and the resulting residue was diluted with DCE and washed with H_2O and brine. The organic layer was dried over Na_2SO_4, filtered and evaporated to dryness under vacuum. The residue was then subjected to FCC (PhMe/EtOAc 8:2) to obtain the pure product as a colorless oil (80%).

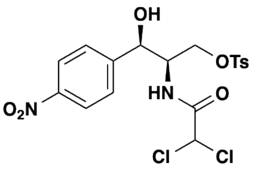

9

R_f (PhMe/EtOAc 8:2): 0.18; **^{1}H-NMR** ($CDCl_3$, 600 MHz): δ 8.16 (d, *J* = 8.6 Hz, 2H), 7.80 (d, *J* = 8.1 Hz, 2H), 7.52 (d, J = 8.6 Hz, 2H), 7.38 (d, J = 8.1 Hz, 2H), 7.17 (d, J = 7.3 Hz, 1H), 6.95 (d, J = 8.5 Hz, 1H), 5.74 (s, 1H), 5.25 (s, 1H), 4.38 – 4.34 (m, 1H), 4.30 (dd, J = 10.2, 7.6 Hz, 1H), 4.06 (dd, J = 10.7, 4.9 Hz, 1H), 2.47 (s, 3H); **^{13}C-NMR** ($CDCl_3$,151 MHz) δ 164.4,

147.7, 145.0, 145.8, 131.9, 130.2, 129.0, 128.2, 128.0, 126.8, 125.3, 123.7, 69.9, 67.1, 65.8, 54.0, 21.7, 21.4; **ESI-MS** (30eV) m/z: 499.0 $[M+Na]^+$

Synthesis of CHL-N_3 (10)

To a solution of the CHL-OTs (1.0 eq) in DMF (0.5 M), NaN_3 (1.1 eq) was added and the reaction mixture was stirred at 100 ^oC overnight. Upon completion of the reaction, the mixture was diluted with AcOEt and washed with H_2O and brine. The organic phase was dried over Na_2SO_4, filtered and evaporated to dryness under vacuum. The residue was subjected to FCC (PhMe/AcOEt 6:4) to obtain the pure product as a yellow oil (77%).

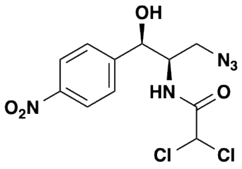

10

R_f (PhMe/EtOAc 6:4): 0.28; **^{1}H-NMR** ($CDCl_3$, 600 MHz): δ 8.19 (d, J = 8.4 Hz, 2H), 7.53 (d, J = 8.4 Hz, 2H), 7.01 (d, J = 8.8 Hz, 1H), 5.17 – 5.05 (m, 2H), 4.203 - 4.18 (m, 1H), 3.71 – 3.74 (m, 1H), 3.61 – 3.54 (m, 1H), 2.93 (s, 1H); **^{13}C-NMR** ($CDCl_3$,151 MHz) δ 164.4, 147.4, 126.7, 123.8, 77.2, 71.4, 66.0, 54.6; **ESI-MS** (30eV) m/z: 370.0 $[M+Na]^+$

Synthesis of CHL-NH_2 (11)

To a solution of the CHL-N_3 (1.0 eq) in THF (0.125 M), Ph_3P (1.2 eq) was added and the reaction mixture was stirred at room temperature overnight. The following day, the mixture was evaporated to dryness under vacuum and the resulting residue was diluted with AcOEt and washed with aqueous HCl 1N twice. The combined aqueous phases were adjusted to pH 8 using aqueous NaOH 2N and was extracted twice with AcOEt. The combined organic phases were washed with H_2O and brine, dried over Na_2SO_4, filtered and evaporated to dryness under vacuum. The residue was subjected to FCC (DCE/MeOH/NH_3 8.7:1.2:0.1) to obtain the pure product **11** as yellow oil (70%).

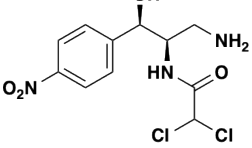

11

R_f (DCE/MeOH/NH_3 8.7:1.2:0.1): 0.16; **^{1}H-NMR** ($CDCl_3$, 600 MHz): δ 8.19 (d, J = 8.4 Hz, 2H), 7.63 (d, J = 8.4 Hz, 2H), 6.27 (s, 1H), 5.19 (d, J = 2.5 Hz, 1H), 4.18-4.16 (m, 1H), 3.86 –

3.83 (m, 1H), 3.68 – 3.61 (m, 2H), 3.34 (p, J = 1.6 Hz, 1H), 1.33 (s, 2H); **^{13}C-NMR** ($CDCl_3$,151 MHz) δ 165.1, 150.2, 147.2, 126.9, 122.7, 69.9, 66.0, 60.8, 57.1; **ESI-MS** (30eV) m/z: 344.0 $[M+Na]^+$

B. Synthesis of modified (*L*)-Lysine building blocks

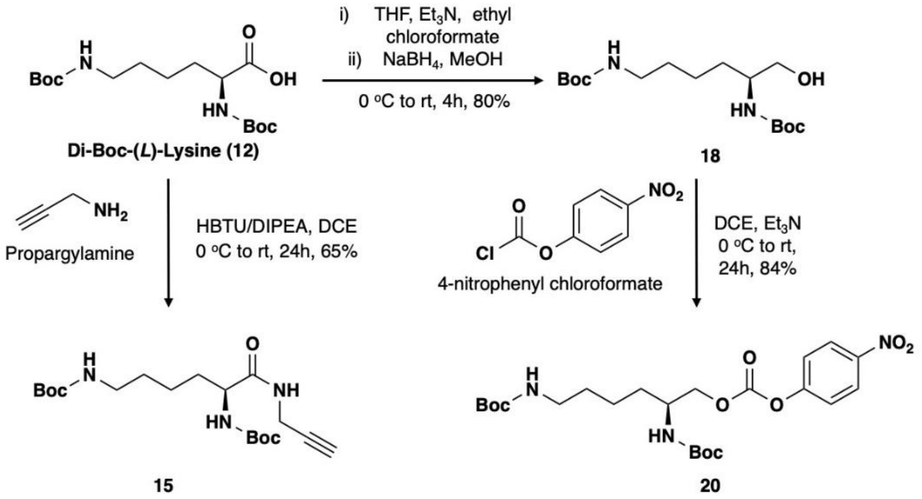

Scheme S2:Synthesis of modified (L)-Lysine building blocks

Synthesis of propargylamide 15

Commercially available Di-Boc-Lys (1.0 eq) was dissolved in DCE (0.7 M) to form a solution. Then, HBTU (1.1 eq) and propargylamine (1.1 eq) were added and the reaction mixture was stirred at 0 °C for 10 min. After 10 min DIPEA (1.0 eq) was added dropwise and the reaction mixture was stirred at room temperature overnight. The next day, the mixture was diluted with DCE and the organic phase was washed with 5% aqueous $NaHCO_3$, H_2O and brine. The organic layer was then dried over Na_2SO_4, filtered and evaporated to dryness under vacuum. The residue was subjected to FCC to obtain the pure product as a colorless oil (65%).

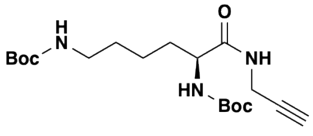

15

R_f (PhMe/EtOAc 6:4): 0.2; **^{1}H-NMR** ($CDCl_3$, 600 MHz): δ 7.32-7.30 (m, 2H), 5.10 (s, 3H), 4.14 - 4.11 (m, 1H), 4.03 – 4.01 (m, 3H), 3.18 (s, 3H), 2.21 – 2.22 (m, 2H), 1.44 (s, 18H); **^{13}C-**

NMR ($CDCl_3$,151 MHz) δ 172.0, 156.6, 136.6, 128.5, 80.2, 79.4, 71.6, 66.6, 54.1, 40.4, 31.9, 29.5, 29.1, 28.3, 22.5; **ESI-MS** (30eV) m/z: 406.3 $[M+Na]^+$

Synthesis of *Di*-Boc-*L*-Lysinol (18)

A solution of commercially available Di-Boc-Lys (1.0 eq) was prepared by dissolving it in THF (0.2 M), and adding Et_3N (1.0 eq) and ethyl chloroformate (1.05 eq). The reaction mixture was then cooled with ice and stirred at 0 °C for 30 min. After 30 min, $NaBH_4$ (3.0 eq) and MeOH were added and the reaction mixture was stirred at room temperature for 3h. Upon completion of the reaction, the mixture was evaporated to dryness under vacuum and and the residue was diluted with AcOEt. The resulting organic phase was then washed with H_2O and brine and the organic layer was dried over Na_2SO_4, filtered and evaporated to dryness under vacuum. Finally, the residue was subjected to FCC to obtain the pure product as a colorless oil (80%).

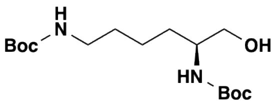

18

R_f (PhMe/EtOAc 8:2): 0.1; **^{1}H-NMR** ($CDCl_3$, 600 MHz): δ 5.00 (d, J = 11.5 Hz, 3H), 3.56 – 3.36 (m, 5H), 3.15 – 3.02 (m, 4H), 1.38 – 1.20 (s, 18H); **^{13}C-NMR** ($CDCl_3$,151 MHz) δ 156.7, 136.6, 128.5, 128.1, 79.7, 66.7, 65.4, 52.6, 40.5, 30.8, 29.7, 22.8; **ESI-MS** (30eV) m/z: 355.3 $[M+Na]^+$

Activation of Di-Boc-*L*-Lysinol (18)

A solution of the derivative **18** (1.0 eq) was prepared by dissolving it in DCE (0.6 M) and adding Et_3N (2.0 eq). The reaction mixture was then cooled with ice and stirred at 0 °C for 30 min. Then 4-nitro-phenylchloroformate (1.2 eq) was added to the reaction mixture, and the mixture was stirred overnight at room temperature. The next day, the reaction mixture was evaporated to dryness under vacuum and the residue was subjected to FCC to obtain the pure product as a yellow oil (84%)

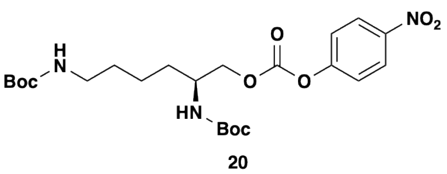

20

R_f (PhMe/EtOAc 9:1): 0.09; **^{1}H-NMR** ($CDCl_3$, 600 MHz): δ 8.28 (d, *J* = 9.1 Hz, 2H), 7.40 (d, *J* = 9.1 Hz, 2H), 5.11 – 5.08 (m, 2H), 4.85 (s, 1H), 4.70 (s, 1H), 4.30 (dd, J = 11.0, 4.2 Hz, 1H), 4.18 (dd, J = 11.0, 5.5 Hz, 1H), 3.95 (s, 1H), 3.73 (d, J = 5.0 Hz, 1H), 3.24 – 3.18 (dq, J = 19.7, 6.8 Hz, 2H), 1.44 (s, 18H); **^{13}C-NMR** ($CDCl_3$,151 MHz) δ 156.6, 155.4, 152.5, 145.4, 125.3, 121.8, 79.9, 71.0, 66.7, 49.1, 40.5 30.8, 29.6, 28.3, 22.7; **ESI-MS** (30eV) m/z: 520.3 $[M+Na]^+$

C. Synthesis of modified (*L*)-Ornithine building blocks

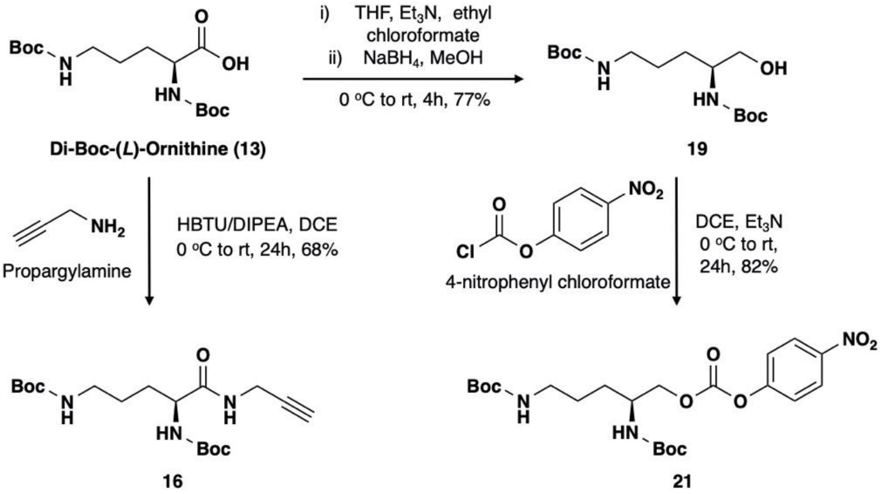

Scheme S3:Synthesis of modified (L)-Ornithine building blocks

Synthesis of propargylamide 16

Commercially available Di-Boc-Orn (1.0 eq) was dissolved in DCE (0.7 M) to form a solution. Then, HBTU (1.1 eq) and propargylamine (1.1 eq) were added and the reaction mixture was stirred at 0 °C for 10 min. After 10 min DIPEA (1.0 eq) was added dropwise and the reaction mixture was stirred at room temperature overnight. The next day, the mixture was diluted with DCE and the organic phase was washed with 5% aqueous $NaHCO_3$, H_2O and brine. The organic layer was then dried over Na_2SO_4, filtered and evaporated to dryness under vacuum. The residue was subjected to FCC to obtain the pure product as a colorless oil (68%).

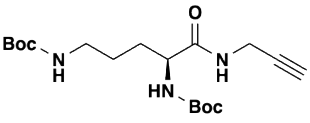

16

R_f (PhMe/EtOAc 7:3): 0.16; **^{1}H-NMR** ($CDCl_3$, 600 MHz): δ 6.96 (s, 1H), 5.32 – 5.22 (m, 1H), 4.31 (s, 1H), 4.04 (dd, J = 5.4, 2.6 Hz, 2H), 3.34 (s, 1H), 3.08 (dt, J = 13.9, 5.7 Hz, 1H), 2.21

(t, J = 2.5 Hz, 1H), 1.84 – 1.79 (m, 1H), 1.64 – 1.51 (m, 3H), 1.46 (s, 18H); **^{13}C-NMR** ($CDCl_3$,151 MHz) δ 172.1, 156.6, 155.8, 129.0, 128.2, 125.3, 79.5, 71.5, 53.0, 39.3, 30.1, 29.0, 28.3, 26.4 ; **ESI-MS** (30eV) m/z: 392.2 $[M+Na]^+$

Synthesis of *Di*-Boc-*L*-Ornithinol (19)

A solution of commercially available Di-Boc-Orn (1.0 eq) was prepared by dissolving it in THF (0.2 M), and adding Et_3N (1.0 eq) and ethyl chloroformate (1.05 eq). The reaction mixture was then cooled with ice and stirred at 0 °C for 30 min. After 30 min, $NaBH_4$ (3.0 eq) and MeOH were added and the reaction mixture was stirred at room temperature for 3h. Upon completion of the reaction, the mixture was evaporated to dryness under vacuum and and the residue was diluted with AcOEt. The resulting organic phase was then washed with H_2O and brine and the organic layer was dried over Na_2SO_4, filtered and evaporated to dryness under vacuum. Finally, the residue was subjected to FCC to obtain the pure product as a colorless oil (77%).

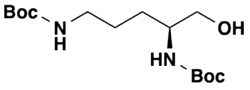

19

R_f (PhMe/EtOAc 1:1): 0.14; **^{1}H-NMR** ($CDCl_3$, 600 MHz): δ 3.67 – 3.50 (m, 3H), 3.14 – 3.08 (m, 2H), 1.60 – 1.48 (m, 3H), 1.42 (s, 18H); **^{13}C-NMR** ($CDCl_3$,151 MHz) δ 156.4, 79.6, 65.3, 52.5, 40.5, 28.4, 26.7.; **ESI-MS** (30eV) m/z: 341.2 $[M+Na]^+$

Activation of *Di*-Boc-*L*-Ornithinol (19)

A solution of the derivative **19** (1.0 eq) was prepared by dissolving it in DCE (0.6 M) and adding Et_3N (2.0 eq). The reaction mixture was then cooled with ice and stirred at 0 °C for 30 min. Then 4-nitro-phenylchloroformate (1.2 eq) was added to the reaction mixture, and the mixture was stirred overnight at room temperature. The next day, the reaction mixture was evaporated to dryness under vacuum and the residue was subjected to FCC to obtain the pure product as a yellow oil (82%).

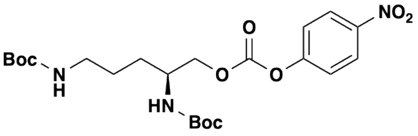

21

R_f (PhMe/EtOAc 9:1): 0.08; **^{1}H-NMR** ($CDCl_3$, 600 MHz): δ 8.28 (d, J = 9.1 Hz, 2H), 7.40 (d, J = 9.1 Hz, 2H), 4.67 – 4.55 (m, 2H), 4.30 (dd, J = 11.0, 4.2 Hz, 1H), 4.20 (dd, J = 11.0, 4.2 Hz, 1H), 3.97 (s, 1H), 3.16 (q, J = 6.3 Hz, 1H), 1.64 – 1.58 (m, 3H), 1.44 (s, 18H); **^{13}C-NMR** ($CDCl_3$,151 MHz) δ 13C NMR (151 MHz, CDCl3) δ 156.0, 155.5, 152.5, 145.5, 125.3, 121.8, 70.9, 49.2, 40.2, 28.7, 28.4, 26.7; **ESI-MS** (30eV) m/z: 506.2 $[M+Na]^+$

D. Synthesis of modified (*L*)-Histidine building blocks

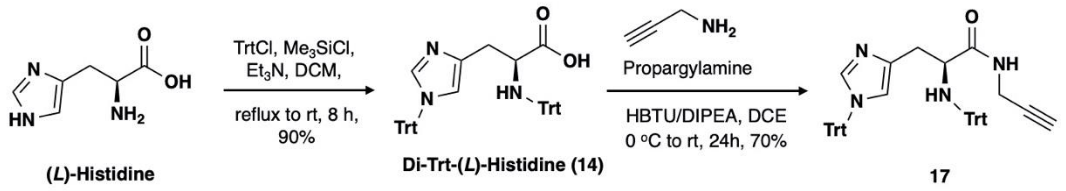

Scheme S4:Synthesis of modified (L)-Histidine building blocks

Trityl-protection of Histidine

To a solution of commercially available *L*-histidine (1.0 eq) in DCM (0.33 M), Me_3SiCl (1.0 eq) was added and the reaction mixture was heated at 30°C for 2 h. Then, it was allowed to cool down to room temperature and a solution of Et_3N (1.0 eq) and TrtCl (2.0 eq) in DCM (0.66 M) was added dropwise. After 8 h the reaction mixture was cooled at 0 °C, MeOH was added and left under stirring at 0 °C for 30 min. Then, it was evaporated to dryness under vacuum and diluted with DCM. The organic phase was washed with 5% aqueous citric acid, H_2O and brine. After being dried over Na_2SO_4, the organic extract was filtered and evaporated to dryness under vacuum. The residue was subjected to FCC (PhMe/AcOEt 1:1) to afford pure **14** as a yellow solid (90%).

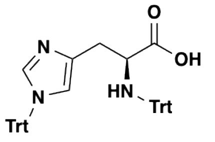

Di-Trt-(*L*)-Histidine (14)

R_f (PhMe/EtOAc 1:1): 0.22; mp 183-185 °C; **^{1}H-NMR** ($CDCl_3$, 600 MHz) δ 7.40 – 7.32 (m, 15H), 7.21 – 7.07 (m, 15H), 6.37 (s, 1H), 3.63 (dd, 1H, J = 7.4, 2.9 Hz), 2.49 – 2.21 (m, 3H), 1.26 (s, 1H); **^{13}C-NMR** ($CDCl_3$,151 MHz) δ 145.5, 129.7, 128.8, 128.4, 128.2, 128.0, 126.6; **ESI-MS** (30eV) m/z: 662.58 $[M+Na]^+$

Synthesis of propargylamide 17

Di-Trt-His (1.0 eq) was dissolved in DCE (0.7 M) to form a solution. Then, HBTU (1.1 eq) and propargylamine (1.1 eq) were added and the reaction mixture was stirred at 0 ^{o}C for 10 min. After 10 min DIPEA (1.0 eq) was added dropwise and the reaction mixture was stirred at room temperature overnight. The next day, the mixture was diluted with DCE and the organic phase was washed with 5% aqueous $NaHCO_3$, H_2O and brine. The organic layer was then dried over Na_2SO_4, filtered and evaporated to dryness under vacuum. The residue was subjected to FCC to obtain the pure product as a pale yellow oil (70%)

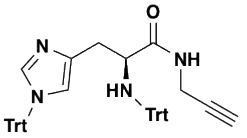

17

R_f (PhMe/EtOAc 8:2): 0.18; **^{1}H-NMR** (Methanol-*d4*, 600 MHz): δ 7.47 (s, 1H), 7.41 – 7.31 (m, 15H), 7.24 – 7.16 (m, 15H), 6.73 (s, 1H), 3.50 (t, J = 6.1 Hz, 1H), 3.35-3.33 (m, 2H), 2.79-2.76 (m, 1H), 2.51 (t, J = 2.6 Hz, 1H), 2.42-2.39 (m, 1H), 2.35 (s, 2H); **^{13}C-NMR** (MeOD ,151 MHz) δ 175.1, 146.0, 142.2, 138.1, 136.3, 129.5, 128.7, 128.5, 127.9, 127.8, 127.8, 127.5, 126.2, 124.9, 120.8, 78.9, 75.5, 71.1, 57.1, 32.9, 28.3; **ESI-MS** (30eV) m/z: 699.3 $[M+Na]^+$

E. Synthesis of derivatives 1-3

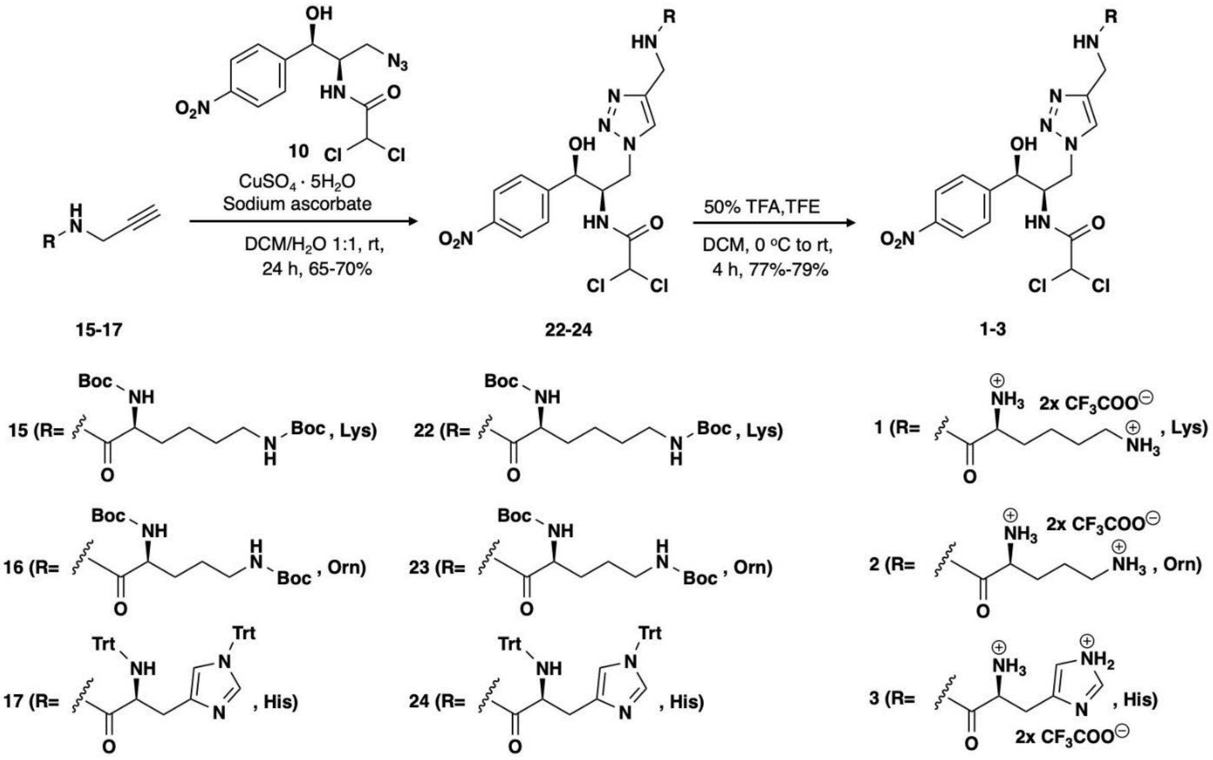

Scheme S5:Synthesis of derivatives 1-3

Synthesis of *N*-protected derivatives 22-24

A solution of the azide **10** (1.0 eq) was prepared by dissolving it in a mixture of DCE and H_2O in a 1:1 ratio (0.02 M). Terminal alkynes **15-17**, sodium ascorbate and copper sulfate pentahydrate (all in 1.0 eq amounts) were then added to the solution. The reaction mixture was stirred at room temperature until the reaction was complete. Upon completion of the reaction, the mixture was diluted with DCE and washed with H_2O. Then the aqueous layer was washed with DCE and the combined organic phases were washed with 5% aqueous $NaHCO_3$, H_2O and brine. The organic layer was dried over Na_2SO_4, filtered and evaporated to dryness under vacuum. Finally, the residue was subjected to FCC to give the pure product as a pale-yellow oil (65-70%)

Derivative 22

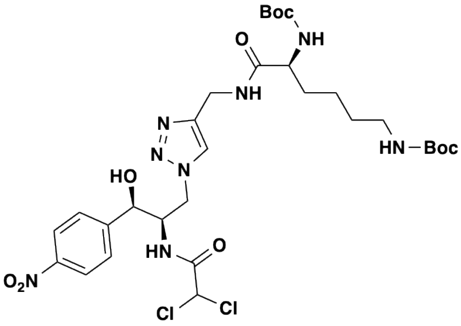

22

R$_f$ (DCE/MeOH 94:6): 0.1; **^{1}H-NMR** ($CDCl_3$, 600 MHz): δ 8.06 (d, J = 7.8 Hz, 4H), 7.53 (s, 3H), 5.94 (s, 1H), 5.03 (s, 2H), 4.77 - 4.66 (m, 4H), 4.13 (s, 1H), 3.09 (s, 3H), 1.75 - 1.62 (m, 4H), 1.36 (s, 18H); **^{13}C-NMR** ($CDCl_3$,151 MHz) δ 164.9, 156.9, 147.9, 147.4, 136.4, 128.6, 128.2, 127.9, 127.0, 125.4, 123.5, 66.7, 65.9, 55.5, 54.5, 40.5, 29.4, 28.3, 22.5; **ESI-MS** (30eV) m/z: 753.3 $[M+Na]^+$

Derivative 23

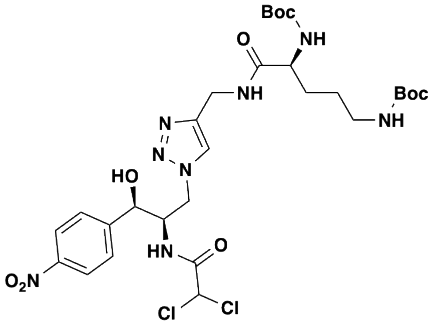

23

R$_f$ (DCE/MeOH 95:5): 0.1; **^{1}H-NMR** ($CDCl_3$, 600 MHz): δ 8.22 (d, J = 8.2 Hz, 2H), 7.73 (s, 1H), 7.53 (d, J = 8.2 Hz, 2H), 5.70 – 5.63 (m, 1H), 5.50 – 5.45 (m, 1H), 4.70 (s, 2H), 4.19 (q, J = 10.5, 5.4 Hz, 2H), 3.77 (t, J = 5.8 Hz, 3H), 3.63 (t, J = 5.8 Hz, 3H), 3.14 – 3.06 (m, 3H), 1.52 (q, J = 8.6, 7.8 Hz, 2H), 1.36 (s, 18H) ; **^{13}C-NMR** ($CDCl_3$,151 MHz) δ 173.2, 171.6, 157.9, 156.0, 148.3, 144.6, 126.5, 124.3, 121,2, 104.5, 80.1, 78.3, 76.8, 71.3, 61.1, 59.6, 42.6, 29.8, 25.8 ; **ESI-MS** (30eV) m/z: 739.3 $[M+Na]^+$

Derivative 24

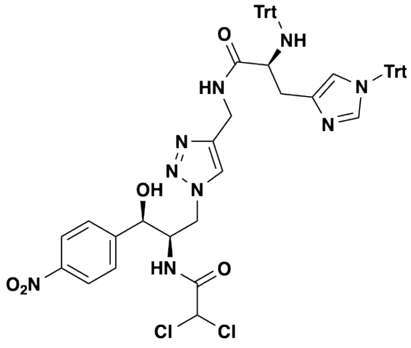

24

Rf (DCE/MeOH 95:5): 0.07; **^{1}H-NMR** (Methanol-*d4*, 600 MHz): δ 8.29 (d, J = 8.2, 2H), 8.17 (d, J = 8.2, 2H), 8.11 – 8.08 (m, 1H), 7.78 (s, 1H), 7.67 – 7.62 (m, 4H), 7.59 – 7.55 (m, 1H), 7.35 – 7.27 (m, 15H), 7.18 – 7.09 (m, 15H), 6.69 – 6.67 (m, 1H), 6.19 (s, 1H), 6.03 (s, 1H), 5.55 – 5.51 (m, 1H), 5.04 (d, J = 1.9 Hz, 1H), 4.98 (d, J = 1.9 Hz, 1H), 3.59 (s, 2H), 2.05 (s, 1H).; **^{13}C-NMR** (151 MHz, MeOD) δ 164.9, 149.4, 149.1, 148.2, 147.3, 145.9, 142.2, 129.4, 128.7, 127.9, 127.5, 127.0, 126.3, 123.7, 122.8,120.9, 78.8, 71.0, 70.4, 65.8, 62.9, 59.4, 55.1, 53.3, 51.5, 50.7, 42.3.; **ESI-MS** (30eV) m/z: 1046.3 $[M+Na]^+$

***N*-Boc or *N*-Trt deprotection of compounds 22-24**

To an ice-cold solution of *N*-Boc or *N*-Trt-protected derivative (1.0 eq) in DCM (0.6 M), a solution of 50% TFA (3.0 eq) in DCM and TFE (2.0 eq) were added. The reaction mixture was stirred for 30 min at 0 ^{o}C and then for 3.5 h at room temperature. Upon completion of the reaction the mixture was triturated with Et_2O and Hex to give the desired product, as a yellow oil in 77-79% yield.

Derivative 1

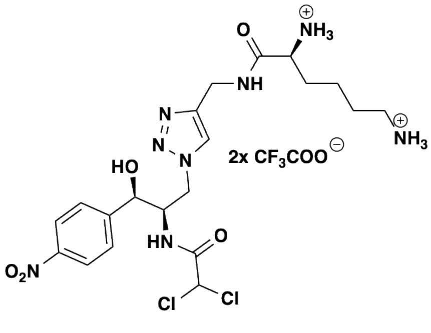

1

Rf (DCM/MeOH/NH_3 8:2:0.2): 0.07

Derivative 2

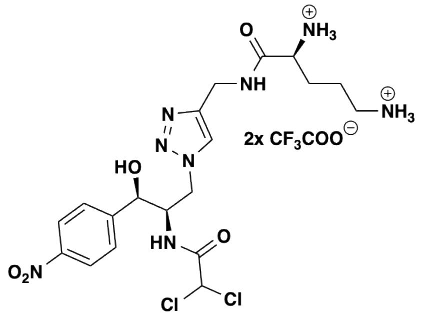

2

R_f (DCM/MeOH/NH_3 8:2:0.2): 0.1

Derivative 3

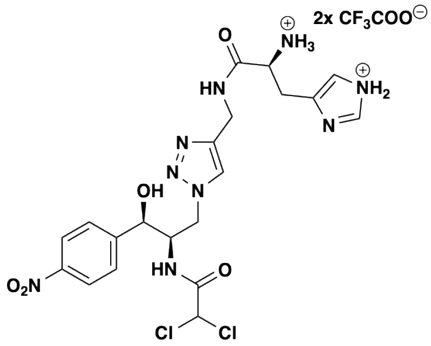

3

R_f (DCM/MeOH/NH_3 8:2:0.2): 0.19

F. Synthesis of amides 4-6, 25-27

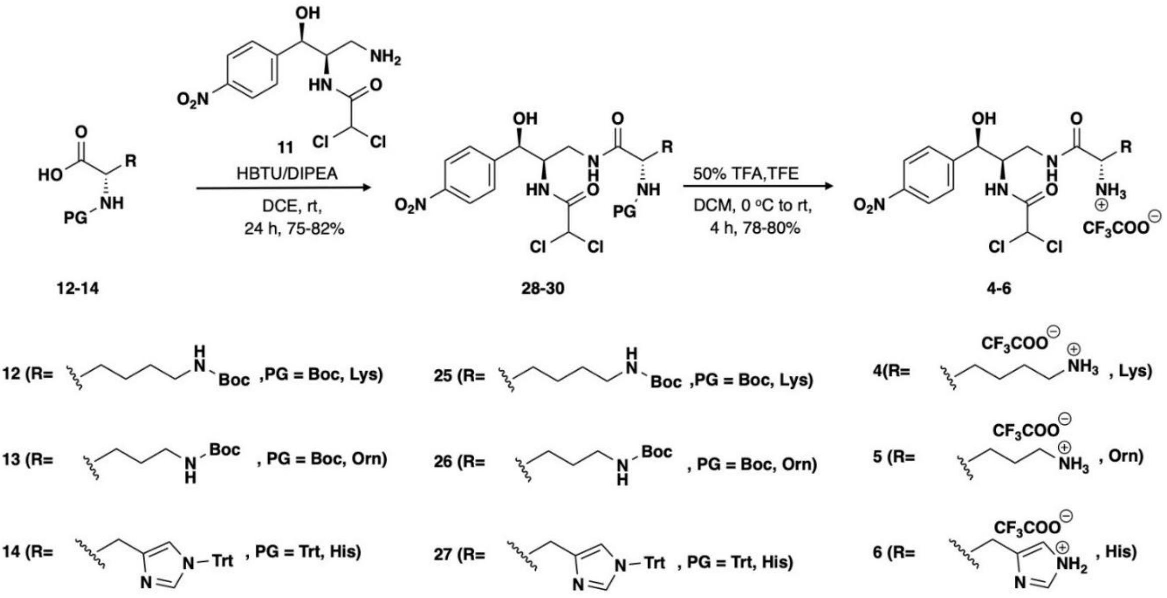

Scheme S6:Synthesis of amides 4-6, 25-27

Synthesis of *N*-protected amides 25-27

To an ice-cold solution of *N*-protected amino acid (1.1 eq) in DCM (0.18 M), DIPEA (1.1 eq) and HBTU (1.1 eq), primary amine **11** (1.0 eq) was added. The reaction mixture was stirred at room temperature and monitored by TLC. Then the mixture was evaporated to dryness under vacuum and the residue thus obtained diluted with AcOEt and washed with 5% aqueous citric acid, H_2O and brine. The organic layer was dried over Na_2SO_4, filtered and evaporated to dryness under vacuum. The residue was subjected to FCC to obtain the pure product as a pale-yellow oil (75-82%)

Amide 25

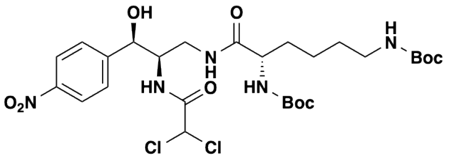

25

R_f (PhMe/EtOAc 6:4): 0.1; **^{1}H-NMR** ($CDCl_3$, 600 MHz): δ 8.24 (d, J = 8.3 Hz, 2H), 7.69 – 7.64 (m, 1H), 7.58 (d, J = 8.3 Hz, 2H), 7.48 – 7.44 (m, 1H), 5.10 – 5.05 (m, 4H), 3.82 – 3.68 (m, 3H), 3.25 – 3.15 (m, 4H), 1.68 (s, 3H), 1.41 (s, 18H) ; **^{13}C-NMR** ($CDCl_3$,151 MHz) δ 170.9, 168.0, 136.5, 132.0, 128.5, 128.1, 124.3, 80.3, 66.7, 52.7, 40.2, 29.7, 29.3, 28.3 ; **ESI-MS** (30eV) m/z: 672.2 $[M+Na]^+$

Amide 26

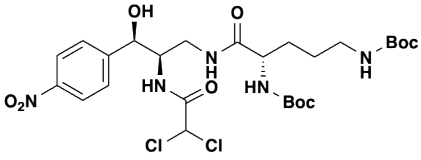

26

R_f (PhMe/EtOAc 7:3): 0.09; **^{1}H-NMR** ($CDCl_3$, 600 MHz): δ 8.23 (d, J = 8.4 Hz, 2H), 7.56 (d, J = 8.4 Hz, 2H), 7.16 (dd, J = 13.0, 7.4 Hz, 1H), 6.09 (d, J = 5.2 Hz, 1H), 5.99 (s, 1H), 4.76 – 4.61 (m, 3H), 4.45 – 4.15 (m, 5H), 3.12 (dq, J = 13.0, 8.5, 6.6 Hz, 5H), 2.35 (s, 1H), 1.42 (s, 18H) ; **^{13}C-NMR** ($CDCl_3$,151 MHz) δ 164.6, 156.1, 148.2, 143.1, 137.8, 129.0, 128.2, 127.6, 125.3, 124.1, 66.0, 53.4, 40.3, 38.1, 31.2, 29.7, 28.2, 26.1, 21.4 ; **ESI-MS** (30eV) m/z: 658.3 $[M+Na]^+$

Amide 27

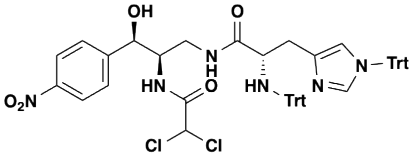

27

R_f (PhMe/EtOAc 8:2): 0.13; **^{1}H-NMR** ($CDCl_3$, 600 MHz): δ 8.23 (d, J = 8.4 Hz, 2H), 7.56 (d, J = 8.4 Hz, 2H), 7.16 (dd, J = 13.0, 7.4 Hz, 1H), 6.09 (d, J = 5.2 Hz, 1H), 5.99 (s, 1H), 4.76 – 4.61 (m, 3H), 4.45 – 4.15 (m, 5H), 3.12 (dq, J = 13.0, 8.5, 6.6 Hz, 5H), 2.35 (s, 1H), 1.42 (s, 18H) ; **^{13}C-NMR** ($CDCl_3$,151 MHz) δ 174.8, 173.5, 165.2, 148.9, 147.3, 145.8, 141.8, 137.4, 132.6, 129.8, 129.5, 128.6, 128.5, 128.4, 128.2, 128.0, 127.8, 127.7, 127.6, 127.5, 127.0, 126.2, 125.5, 124.9, 122.8, 122.6, 121.3, 121.0, 76.2, 72.3, 71.0, 70.6, 70.3, 69.9, 68.4, 65.8, 65.7, 64.2, 63.5, 61.0, 56.4, 56.2, 54.2, 54.0, 50.88, 41.0, 37.5, 32.6, 20.2 ; **ESI-MS** (30eV) m/z: 965.3 $[M+Na]^+$

***N*-Boc or *N*-Trt deprotection of amides 25, 26 and 27**

To an ice-cold solution of *N*-Boc or *N*-Trt-protected derivative (1.0 eq) in DCM (0.6 M), a solution of 50% TFA (3.0 eq) in DCM and TFE (2.0 eq) were added. The reaction mixture was stirred for 30 min at 0 ^{o}C and then for 3.5 h at room temperature. Upon completion of the reaction the mixture was triturated with Et_2O and Hex to give the desired product, as a yellow oil in 78-80% yield.

Amide 4

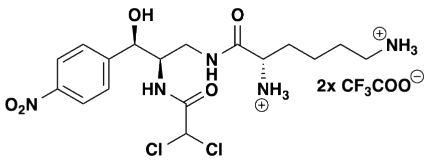

4

R_f (DCM/MeOH/NH_3 9:1:0.2): 0.06

Amide 5

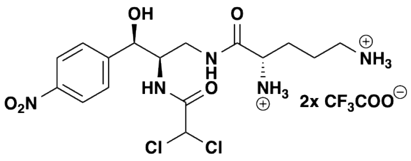

5

R_f (DCM/MeOH/NH_3 8:2:0.2): 0.07

Amide 6

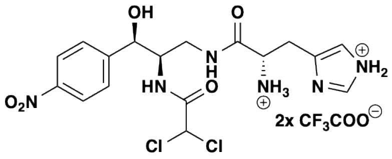

6

R_f (DCM/MeOH/NH_3 8:2:0.2): 0.05

G. Synthesis of carbamates 7-8, 28-29

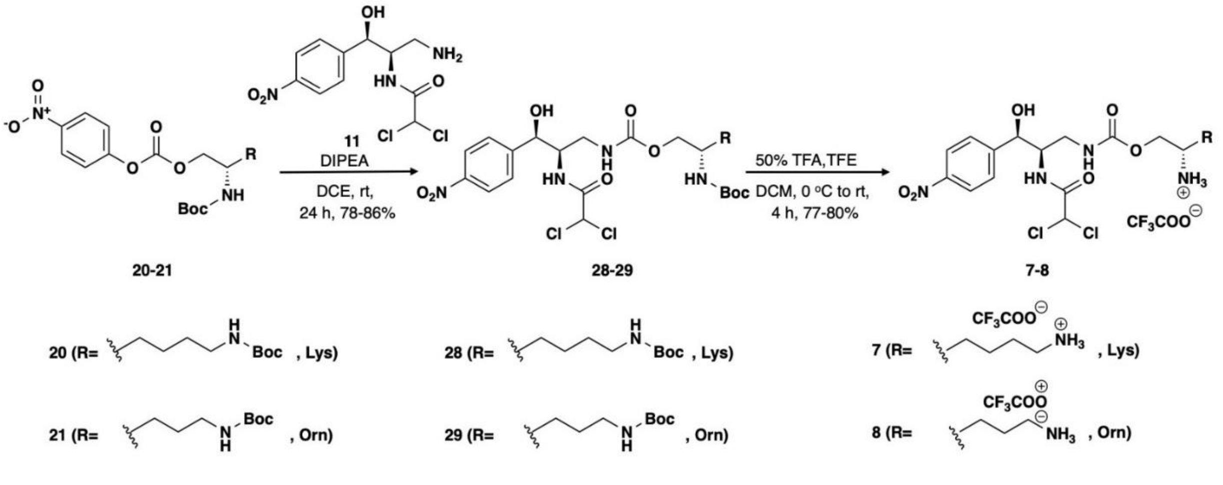

Scheme S7:Synthesis of carbamates 7-8, 28-29

Synthesis of *N*-protected carbamates 28-29

To an ice-cold solution of *N*-protected amino acid (1.1 eq) in DCM (0.18 M) and DIPEA (1.1 eq), primary amine **11** (1.0 eq) was added. The reaction mixture was stirred at room temperature and monitored by TLC. Then, the mixture was evaporated to dryness under vacuum and the residue thus obtained diluted with AcOEt and washed with 5% aqueous $NaHCO_3$, H_2O and brine. The organic layer was dried over Na_2SO_4, filtered and evaporated to dryness under vacuum. The residue was subjected to FCC to give the pure product as a pale-yellow oil (78-86%)

Carbamate 28

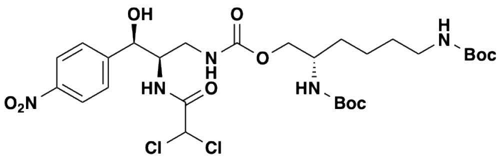

28

R_f (PhMe/EtOAc 3:7): 0.11; ^{1}**H-NMR** ($CDCl_3$, 600 MHz): δ 8.25 (d, J = 8.5 Hz, 2H), 7.66 – 7.63 (m, 1H), 7.58 (d, J = 8.5 Hz, 2H), 7.48 – 7.45 (m, 1H), 7.31 – 7.29 (m, 3H), 5.36 (d, J = 5.9 Hz, 1H), 5.08 (s, 2H), 5.01 (s, 1H), 4.70 (s, 1H), 4.07 – 4.04 (m, 2H), 3.79 – 3.75 (m, 2H), 3.51 – 3.41 (m, 2H), 3.18 (q, J = 7.2 Hz, 2H), 2.37 – 2.11 (m, 1H), 1.75 (s, 2H), 1.40 (s, 18H); ^{13}**C-NMR** ($CDCl_3$,151 MHz) δ 158.2, 157.0, 155.8, 148.2, 136.5, 132.0, 128.5, 128.1, 127.9, 126.4, 124.2, 79.6, 78.8, 76.8, 66.7, 60.5, 49.5, 43.5, 40.4, 30.6, 29.5, 28.4, 22.5 ; **ESI-MS** (30eV) m/z: 702.2 $[M+Na]^+$

Carbamate 29

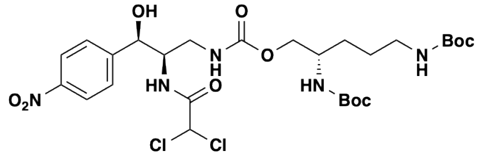

29

Rf (PhMe/EtOAc 3:7): 0.09; **^{1}H-NMR** ($CDCl_3$, 600 MHz): δ 8.26 (d, J = 8.4 Hz, 2H), 7.66 – 7.63 (m, 1H), 7.60 (d, J = 8.4 Hz, 2H), 7.57 – 7.53 (m, 1H), 7.48 7.45 (m, 1H), 6.54 (s, 1H), 5.89 (s, 1H), 5.38 (d, J = 5.7 Hz, 1H), 4.72 – 4.76 (m, 2H), 4.08 – 4.02 (m, 2H), 3.86 – 3.77 (m, 2H), 3.60 – 3.44 (m, 2H), 3.14 – 3.10 (m, 2H), 1.70 (s, 3H), 1.41 (s, 18H).; **^{13}C-NMR** ($CDCl_3$,151 MHz) δ 176.7, 165.5, 152.6, 148.2, 132.6, 132.1, 128.6, 126.4, 126.4, 124.2, 78.8, 60.6, 43.5, 40.2, 28.4, 26.6.; **ESI-MS** (30eV) m/z: 688.3 $[M+Na]^+$

***N*-Boc deprotection of carbamates 28 and 29**

To an ice-cold solution of Boc-protected carbamates (1.0 eq) in DCM (0.6 M), a solution of 50% TFA (3.0 eq) in DCM and TFE (2.0 eq) were added. The reaction mixture was stirred for 30 min at 0 °C and then for 2.5 h at room temperature. Upon completion of the reaction the mixture was triturated with Et_2O and Hex to give the desirable product, as a yellow oil in 77%-80% yield.

Carbamate 7

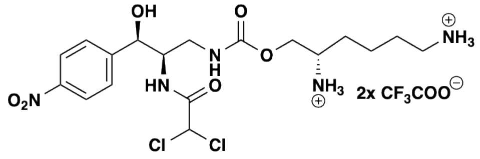

7

Rf ($DCM/MeOH/NH_3$ 9:1:0.1): 0.2

Carbamate 8

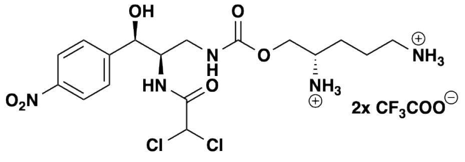

8

R_f (DCM/MeOH/NH_3 9:1:0.1): 0.18
